# Effect of *Lippia alba* (Mill.) N.E. Brown Essential Oil on the Human Umbilical Artery

**DOI:** 10.3390/plants11213002

**Published:** 2022-11-07

**Authors:** Alex S. Borges, Carla M. S. Bastos, Debora M. Dantas, Cícera G. B. Milfont, Guilherme M. H. Brito, Luís Pereira-de-Morais, Gyllyandeson A. Delmondes, Renata E. R. da Silva, Emanuel Kennedy-Feitosa, Francisco P. A. Maia, Clara M. G. Lima, Talha Bin Emran, Henrique Douglas M. Coutinho, Irwin Rose A. Menezes, Marta R. Kerntopf, Gianluca Caruso, Roseli Barbosa

**Affiliations:** 1 Biological Chemistry Department, Postgraduate Program in Biological Chemistry, Pimenta Campus, Regional University of Cariri, Crato 63105-010, Ceará, Brazil; 2 Biological Sciences Department, Physiopharmacology of Excitable Cells Laboratory, Pimenta Campus, Regional University of Cariri, Crato 63105-010, Ceará, Brazil; 3 Nursing Collegiate, Petrolina Campus, Federal University of The San Francisco Vale, Petrolina 56304-205, Pernambuco, Brazil; 4 Health Science Department, Morphophysiopharmacology Laboratory, Federal Rural University of Semiarid, Mossoró 59625-900, Rio Grande do Norte, Brazil; 5 CECAPE College, Juazeiro do Norte 63024-015, Ceará, Brazil; 6 Department of Food Science, Federal University of Lavras, Lavras 37200-900, Minas Gerais, Brazil; 7 Department of Pharmacy, BGC Trust University Bangladesh, Chittagong 4381, Bangladesh; 8 Department of Pharmacy, Faculty of Allied Health Sciences, Daffodil International University, Dhaka 1207, Bangladesh; 9 Department of Agricultural Sciences, University of Naples Federico II, 80055 Naples, Italy

**Keywords:** essential oil, *Lippia alba*, human umbilical artery, vasorelaxant effect

## Abstract

*Lippia alba* is popularly known as lemon balm, with its essential oil (EO) cited for displaying antimicrobial, sedative, and vasorelaxant effects. Yet, its action on isolated human vessels has not been described in the literature. Thus, we evaluated the vasorelaxant effect of essential oil of *L. alba* (EOLa) on human umbilical arteries (HUA) isolated in organ baths. HUA rings were isolated, subjected to contractions induced by potassium chloride (KCl), serotonin (5-HT), or histamine (HIST) to record the isometric tension, and then treated with EOLa (30–1000 µg/mL). The EOLa showed a more prominent inhibitory effect on the pharmacomechanical coupling contraction via HIST with an EC_50_ value of 277.1 ± 8.5 µg/mL and maximum relaxant effect at 600 µg/mL. The addition of tetraethylammonium (TEA) or 4-aminopyridine (4-AP) in HUA preparations did not inhibit EOLa total relaxant effect at 1000 µg/mL. In the presence of gliblenclamide (GLI), the oil relaxed the HUA rings by 90.8% at maximum concentration. The EOLa was also investigated for its effects on voltage-operated calcium channels (VOCCs), where the HUA preincubation with this oil at 1000 μg/mL inhibited BaCl_2_ (0.1–30 mM)-induced contractions. This study demonstrates for the first time that EOla has a vasorelaxant effect on HUA and its particular blockade of VOCCs.

## 1. Introduction

Essential oils (EOs) are aromatic complexes extracted from plants that contain a mixture of volatile organic compounds, which are present in the various organs of plants (roots, barks, leaves, flowers and fruits). These oils have a variety of pharmacological applications, as they have important biological properties for the plants themselves, for human beings and for other animals [1].

The species *Lippia alba* (Mill.) N.E. Brown belonging to the Verbenaceae family is found in Central America, South America, Africa, and Asia [2]. It is popularly known as lemon herb balm, Brazilian lemon balm, False-Melissa, Carmelite lemon balm, common lemon balm tea, field lemon balm and bush matgrass, among others [3,4]. The essential oil of this species is described as antibacterial [5,6,7], antifungal [8,9,10,11], anxiolytic, anticonvulsant [12,13], anesthetic [14,15,16], and smooth muscle relaxant [17,18,19,20]. Moreover, in folk medicine, this species is used to control blood pressure, as a calmant, and to treat respiratory diseases [21,22,23].

Searching for natural products that promote vasoactivity makes possible the discovery of therapeutic agents with a better efficacy and safety profile to treat disorders that cause hypertensive states. For example, pre-eclampsia is a hypertensive syndrome affecting the gestational period that increases the vascular umbilical resistance, resulting in a health risk to both the mother’s and the baby’s life [24,25].

The prevalence of pregnant women with hypertensive syndromes continues to increase, on the other hand, the most prescribed antihypertensive drugs are not recommended during pregnancy. There are few medications that are prescribed during pregnancy to control pre-eclampsia and eclampsia. However, the side effects of these are accounted for increasing the risk of fetal growth restriction and neonatal bradycardia, among other effects like depression for the mother [26,27].

Studies that prove the vasorelaxant potential of EOLa are only available on rodent tissue models [17,18,19,20]. Moreover, few are the studies that demonstrate the potential activity of natural products in human umbilical artery smooth musculature [28,29]. The use of human umbilical cord vessels as a model for screening substances with vasorelaxant properties is of great relevance for gestational hypertensive disorders, in addition to other cardiovascular diseases [30,31]. Herein, we show for the first time the vasorelaxant effect of *L. alba* essential oil on isolated human umbilical artery.

## 2. Results

### 2.1. Effect of the Essential Oil of Lippia alba on Basal Vascular Tone in Human Umbilical Artery

**Figure 1:**
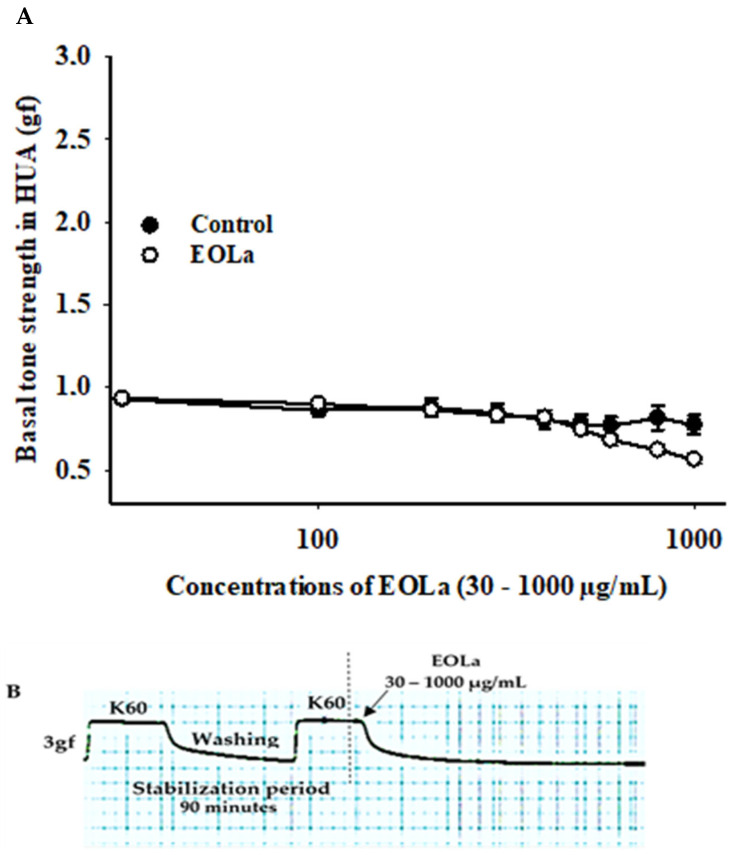
Effect of EOLa on basal vascular tone in HUA. (**A**) “Log-normal” plot for the effect of EOLa (30–1000 µg/mL) on basal HUA tone. The values are expressed as the mean ± SEM; n = 6 (*p* < 0.001, one-way ANOVA). (**B**) Original register in LabChart Pro software for relaxant effect of EOLa on vascular basal tone in HUA.

EOLa changed the final basal tone (*p* < 0.017), being significant at 1000 µg/mL compered to the control (*p* < 0.001) (Figure 1).

### 2.2. Relaxant Effect of EOLa on Contractions Induced by KCl (60 mM), 5-HT (10 µM), and HIST (10 µM) in HUA

**Figure 2:**
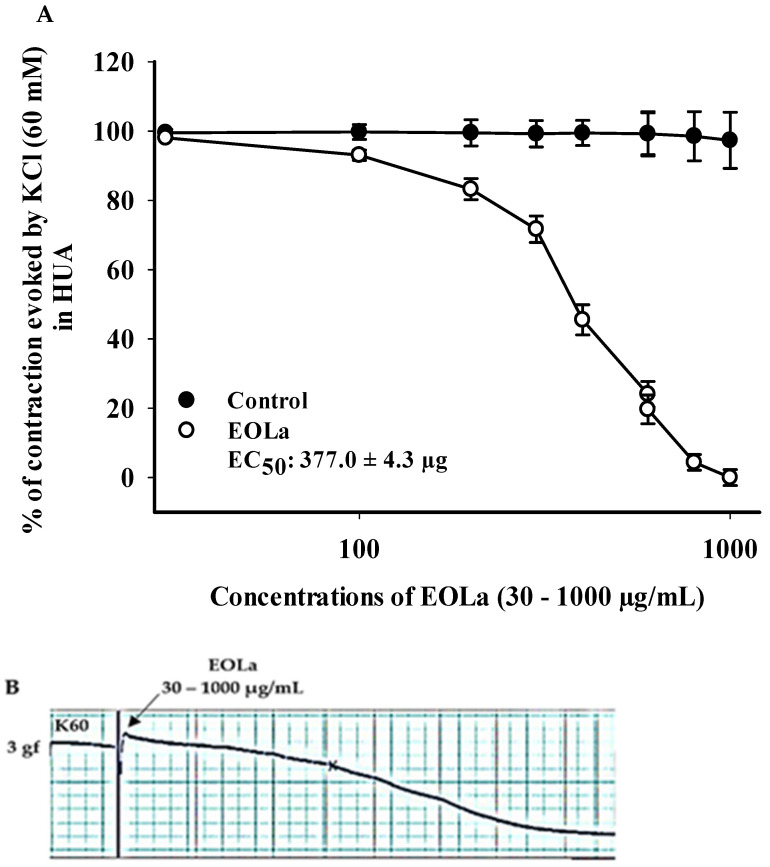
Relaxing effect of EOLa and evaluation of K60 electromechanical coupling in HUA. (**A**) “Log-normal” plot for EOLa (30–1000 μg/mL) on contractions induced by potassium chloride in HUA; values are expressed as the mean ± SEM; n = 6 (*p* < 0.017, one-way ANOVA). (**B**) Original registration made by the LabChart Pro software for the relaxing effect of EOLa on the electromechanical coupling induced by K60 in HUA.

When evaluating the effect of EOLa on electromechanical coupling produced by KCl at 60 mM (K60), vasorelaxant activity was observed from 100 µg/mL (*p* < 0.017, one-way ANOVA) with EC_50_ at 377.0 ± 4.3 µg/mL (Figure 2).

**Figure 3:**
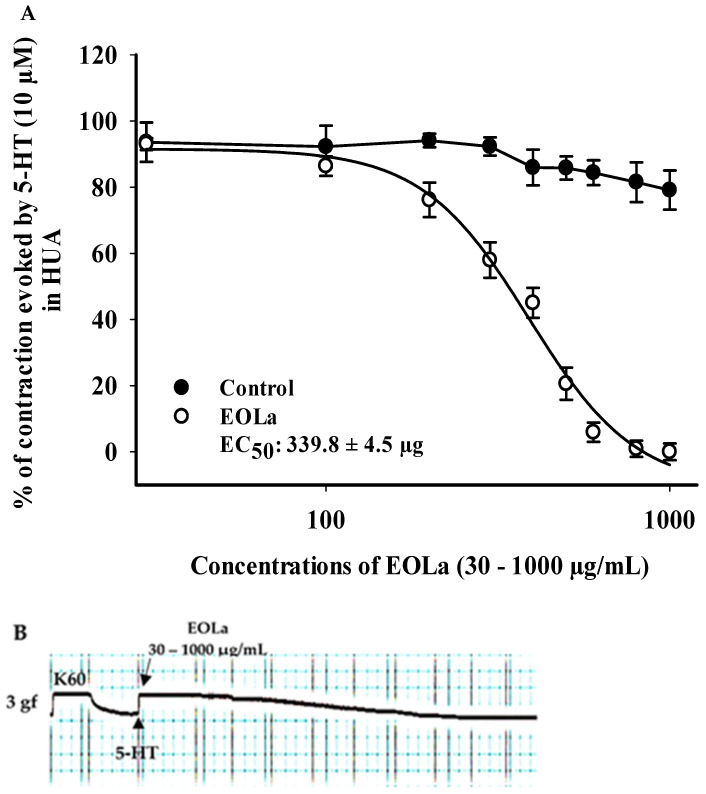
Relaxant effect of EOLa and evaluation of pharmacomechanical coupling by 5-HT (10 µM) in HUA. (**A**) “Log-normal” plot for the effect of EOLa (30–1000 μg/mL) on serotonin-induced contractions in HUA; values are expressed as the mean ± SEM; n = 6 (*p* < 0.01, one-way ANOVA). (**B**) Original recording in LabChart Pro software for relaxant effect of EOLa on the pharmacomechanical coupling induced by 5-HT in HUA.

**Figure 4:**
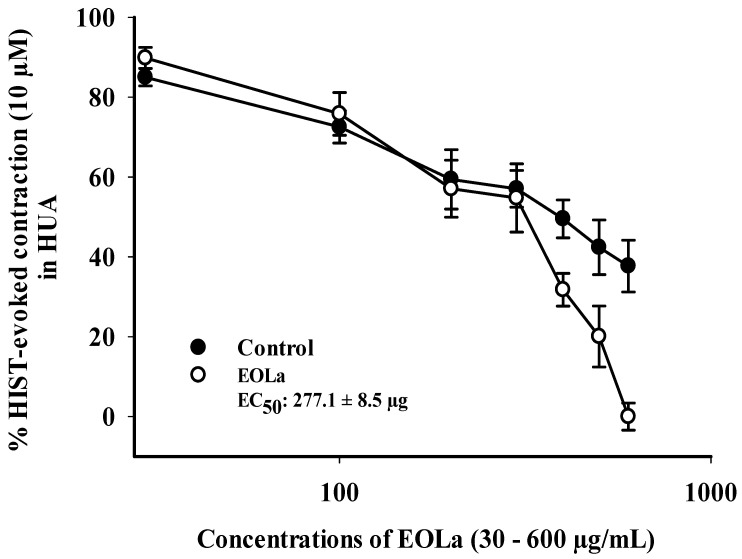
Relaxant effect of EOLa (30–1000 μg/mL) and evaluation of pharmacomechanical coupling by HIST (10 µM) in HUA. “Log-normal” plot for the effect of EOLa on contractions induced by histamine in HUA. Values are expressed as the mean ± SEM; n = 6 (*p* < 0.01, one-way ANOVA).

**Figure 5:**
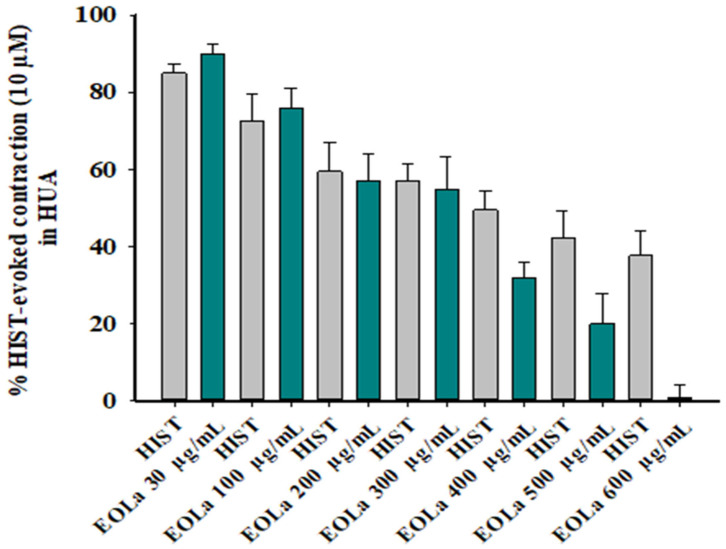
Relaxant effect of EOLa (30–600 μg/mL) and evaluation of pharmacomechanical coupling by HIST (10 µM) in HUA. Concentration–response bar graph for the effect of EOLa on contractions induced by histamine in HUA. Values are expressed as the mean ± SEM; n = 6 (*p* < 0.01, one-way ANOVA).

When evaluating the pharmacomechanical pathway, it was observed that the EOLa promoted total blockage of 5-HT (10 μM)- and HIST (10 μM)-induced contractions in HUA rings. The blockade proved to be significant starting at a concentration of 200 μg/mL (*p* < 0.002, one-way ANOVA), with an EC_50_ value of 339.8 ± 4.5 µg/mL in experiments using the 5-HT agonist (Figure 3). When the HIST agonist was used, we observed a significant EOLa effect starting at a concentration of 200 μg/mL (*p* < 0.001 one-way ANOVA), with EC_50_ 277.1 ± 8.5 µg/mL, and total relaxation of the HUA rings at an EOLa concentration of 600 µg/mL. The control preparations—contracted using HIST—presented significant degradation; the effect of EOLa is also represented in the bar graph (Figure 4 and Figure 5).

### 2.3. Relaxant Effect of EOLa on High Conductance K^+^ Channels Activated by Ca^2+^ (BKCa), Voltage-Operated K^+^ Channels (Kv) and ATP-Sensitive K^+^ Channels (K_ATP_)

**Figure 6:**
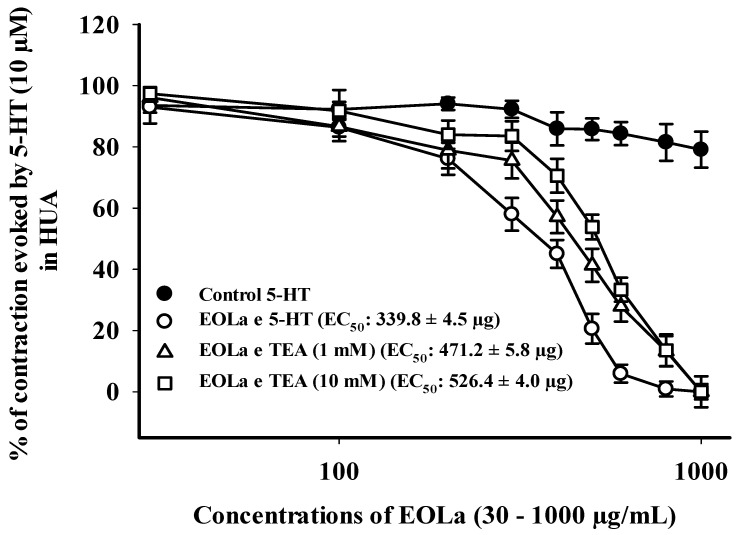
Relaxant effect of EOLa (30–1000 μg/mL) and assessment of the participation of high conductance K^+^ channels activated by Ca^2+^ (BKCa) and voltage-operated K^+^ channels (K*v*) in HUA. “Log-normal” plot for EOLa effect on contractions evoked by 5-HT (10 µM) in HUA rings preincubated in TEA (1 mM or 10 mM). Values are expressed as the mean ± SEM; n = 6 (*p* < 0.05, one-way ANOVA).

**Figure 7:**
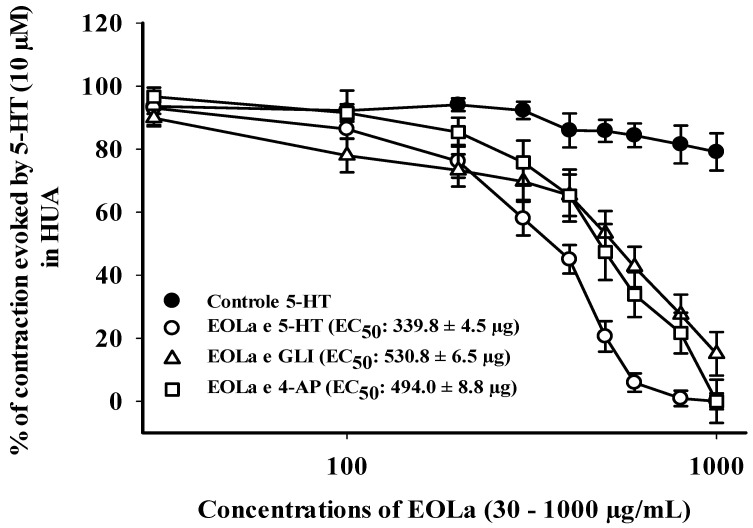
Relaxant effect of EOLa (30–1000 μg/mL) and evaluation of the participation of voltage-operated K^+^ channels (*Kv)* and ATP-sensitive K^+^ channels (K*_ATP_)* in HUA. “Log-normal” plot for the effect of EOLa on stimulated contractions using 5-HT (10 µM) in HUA rings preincubated with 4-AP (1 mM) or GLI (10 µM). Values are expressed as the mean ± SEM; n = 6 (*p* < 0.05, one-way ANOVA).

EOLa (30–1000 µg/mL) relaxed 100% of the preincubated HUA preparations with TEA. The EOla initial significant concentration was 200 µg/mL for both TEA concentrations used: TEA (1 mM)—*p* < 0.023, one-way ANOVA; EC_50_ of 471.2 ± 5.8 µg/mL; TEA (10 mM)—*p* < 0.017, one-way ANOVA; EC_50_ of 526.4 ± 4.0 µg/mL (Figure 6). The EC_50_ values for EOla obtained in preincubated HUA rings with 4-AP (1 mM) or GLI (10 µM) preparations were 494.0 ± 8.8 µg/mL and 530.8 ± 6.5 µg/mL, respectively. The concentrations statistically significant started at 200 µg/mL and 400 µg/mL (*p* < 0.001, one-way ANOVA), respectively. In the presence of 4-AP, EOLa achieved 100% of relaxation with a higher EC_50_ value. However, in the presence of GLI, EOLa performed 90.8% of relaxation, demonstrating some participation of K*_ATP_* channels in its vasodilatory response (Figure 7).

### 2.4. Effect of EOLa on Voltage-Operated Ca^2+^ Channels (VOCCs)

**Figure 8:**
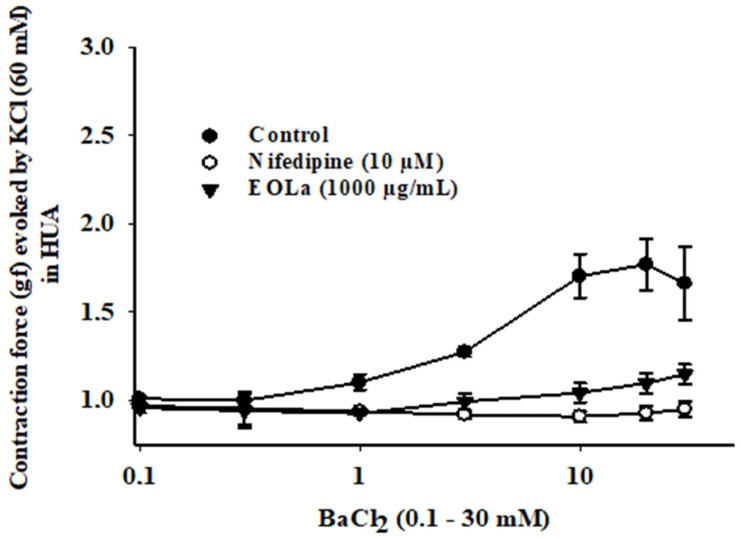
Relaxant effect of EOLa (1000 µg/mL) and assessment of participation of voltage-operated calcium channels (VOCCs) in HUA. Control: “Log-normal” plot for the contractile effect by cumulative addition of BaCl_2_ (0.1–30 mM) in HUA preparations. Nifedipine: Graph for inhibitory effect of nifedipine (10 µM) on induced contraction by cumulative addition of BaCl_2_ (0.1–30 mM); positive control. EOLa: Graph for the contraction-blocking effect of EOLa (1000 µg/mL) on accumulative addition of BaCl_2_ (0.1–30 mM). Values are expressed as the mean ± SEM; n = 6 (*p* < 0.05, one-way ANOVA).

Preincubation with EOLa (1000 µg/mL) reduced the contraction response of HUA rings to BaCl_2_ (0.1–30 mM). This reduction was similar to that observed in rings preincubated with nifedipine (10 µM), a selective L-type VOCCs (Figure 8). Table 1 presents the main findings of this study, with EC_50_ values of the EOLa effect in the presence of each contractile agonist, as well as K^+^ channel blockers.

## 3. Discussion

*Lippia alba* is well known for its essential oils, and various concentrations of compounds have been isolated and identified deriving from different parts of the plant. Terpenes and its derivatives are the major constituents, generally known for their applications as flavors and fragrances. The structures of these compounds are variable, and their classification is based on the number of carbon chains and chemical function presented. The main types of terpenes found in *L. alba* are the monoterpenes and sesquiterpenes [2].

This is the first study investigating the effect of EOLa on the contractility of human umbilical arteries. EOLa under study has as major constituents the monoterpenoid citral (a mixture of two aldehyde geometric isomers, E-geranial (41.81%) and Z-neral (34.11%)), the monoterperne 1-limonene (a cyclic unsaturated hydrocarbonate), and the monoterpenoid carvone (a cyclic unsaturated oxygenated ketone).

Published data with different rat isolated smooth muscles show that EOLa has a relaxant effect with possible main mechanism of action by blocking L-type voltage-operated Ca^2+^ channels (VOCCs) [17,19,20,32], thus supporting our results. Regarding their various electrophysiological and pharmacological properties, VOCCs were initially classified as types L, N, P/Q, R, and T [33]. L-type VOCCs are designated as CaV1.1–1.4, N-type VOCCs are designated as CaV2.2, the P/Q type is designated as CaV 2.1, and type R is designated as CaV2.3, which are insensitive to dihydropyridine and activated by high voltage; lastly, T-type channels CaV3.1–3.3 are activated by low voltage [34,35]. In smooth muscle cells, L-type VOCCs are the most studied, since they have great importance in contraction processes [36,37]. In this study, EOLa presented a response similar to the positive control nifedipine (10 µM), a selective L-type VOCC blocker. Hence, we believe that these channels are prominently involved in the relaxant effect of EOLa on the electromechanical coupling contraction.

Carvalho et al. [17], used EOLa, citral, or 1-limonene on rat trachea demonstrating that the oil and citral alone had a similar relaxant effect over tracheal smooth muscle contracted by K60 electromechanical coupling, including with blockade of VOCCs similar to the positive control nifedipine (1 µM), thus inhibiting contractions by BaCl_2_, as well as by acetylcholine on pharmacomechanical coupling. On the other hand, 1-limonene did not display great relaxant effect, while also not being unable to inhibit contractions evoked by BaCl_2_.

Pereira-de-Morais et al. [19], used EOLa, citral, or 1-limonene on rat uterus, observing that the oil or each of its main terpene constituents produced a relaxant effect on contractions induced by various contractile agents. For both electromechanical and pharmacomechanical coupling via oxytocin, serotonin, and acetylcholine, all had the same efficacy on total contraction inhibition at the same concentration. However, analyzing the inhibitory concentrations (IC_50_) used, there was a difference amongst them, with EOLa having lower IC_50_ values than citral and 1-limonene, suggesting that the other compounds that make up a smaller proportion of the composite essential oil may be active, possibly contributing to the relaxant effect.

Seres-Bokor et al. [38] suggested an alternative mechanism for the relaxing effect of citral on rat uterus. A 14 day treatment in vivo with this monoterpenoid induced an increase of aquaporin channel (AQP5) expression in uterine smooth muscle, reducing hypertonic stress activation on the transient receptor potential cation channel receptor type 4 (TRPV4).

Silva et al. [20] used OELa and citral on rat aorta artery, corroborating the findings of our study, since the effect observed for EOLa was quite significant in the electromechanical pathway, presenting even greater effect in the thoracic artery (EC_50_: 83.30 μg/mL) than in the HUA (EC_50_: 377.0 ± 4.3 µg/mL). However, these differences can be explained by the fact that the blood vessels of experimental animals and humans, specifically the HUA, are distinct from each other in terms of physiology, receptor types, and activity of specific signaling pathways [39].

Maynard et al. [32] used EOLa on rat mesenteric artery rings, showing an endothelium-independent vasorelaxant effect on both electromechanical by KCl (80 mM) and pharmacomechanical coupling by phenylephrine (1 µM), possibly due to an inhibition on the Ca^2+^ influx through the L-type VOCCs. The EO applied in this study also has the constituent citral in majority.

Few studies have reported the vasorelaxant activities of plant natural products in human umbilical vessels. Among them, one that shows *Cymbopogon citratus* extract, rich in polyphenols (chlorogenic acid, iso-orientin, and swertiajaponin), is able to inhibit vasoconstriction induced by the thromboxane A2 receptor agonist U46619 [40]. Another with octylmethoxycinnamate that activates soluble guanylate cyclase and inhibits VOCCs, subsequently causing vasorelaxation [41]. This mechanism was also observed for genistein on HUA, a natural phytoestrogen belonging to the isoflavone group [29].

It is known that HUA vascular responsiveness occurs more effectively to contracting agents than to relaxing agents [42]. Since umbilical blood vessels are not innervated, control of umbilical blood flow depends on vasoactive substances released locally or already existing in circulation such serotonin, histamine, oxygen, nitric oxide (NO), thromboxane, and ions (e.g., calcium and potassium) [43,44,45,46]. Furthermore, 5-HT and HIST are the most potent vasoconstrictors in umbilical human arteries, and increased release of these mediators, as well as increased sensitivity in HUA to these agents, has already been related to certain pathological processes disturbing the umbilical cord circulation (e.g., preeclampsia) [47,48]. Thus, mechanisms that regulate HUA smooth muscle contractility are of paramount importance for placental exchange of both gases and nutrients with the fetus.

The EOLa was able to relax the smooth musculature in HUA with greater potency on 5-HT-evoked contractions (EC50 = 339.8 µg/mL ± 4.5 µg/mL) than on K60-evoked contractions (EC50 = 377.0 µg/mL ± 4.3 µg/mL). An even more promising relaxing effect of EOLa was demonstrated by the histaminergic coupling protocol, with an EC_50_ of 277.1 ± 8.5 μg/mL. This difference suggests that it also acts on distal targets of intracellular cascades activated on the pharmacomechanical pathway. Silva et al. [20] demonstrated that EOLa and citral equally inhibited contraction evoked by phorbol-12,13-dibutyrate—an activator of intracellular calcium-binding proteins, such as protein-kinase C (PKC), which are active in contraction of smooth muscle—in rat aorta arteries isolated in zero-calcium medium.

Potassium channels play a key role in membrane potential regulation and cell excitability, with smooth muscle contraction dependent on the equilibrium between increasing K^+^ ion conductance, leading to hyperpolarization, and decreasing K^+^ conductance (leading to depolarization) [49,50]. In smooth muscle, the basal tone can be regulated by several subtypes of K^+^ channels. Using TEA (10 mM) as a nonselective K^+^ channel blocker, including blockade of large conductance K^+^ channels activated by Ca^2+^ (BKCa), and TEA (1 mM), as a more sensible blockade on voltage-operated K^+^ channels (K*v*), an increase in the EC_50_ values was observed; however, the oil promoted total relaxation at the maximum concentration used. A similar effect was seen for the monoterpenoid carveol on human umbilical arteries [28].

Comparing the relaxing potential of EOla in the presence of 4-AP (a K*v* blocker) or GLI (a blocker for ATP-sensitive K^+^ channels (K*_ATP_*)) we observed an expressed increase for EC_50_ value when using GLI. In addition, there was a 9.2% decrease in the relaxant potential of the EOLa at maximum concentration. This indicates the influence of these pathways, especially K*_ATP_*, on the relaxant effect of EOLa in human umbilical arteries.

Potassium channels activated by intracellular metabolites such as ATP can hyperpolarize the membrane, inhibiting Ca^2+^ influx by blocking VOCCs and, consequently, favoring relaxation of smooth muscle cells. K*_ATP_* is designed to have the ability to decrease its activity when the intracellular levels of ATP increase. This activity can also be modulated by ATP-independent signaling pathways [51]. In a study carried out with labdane-302, a diterpene isolated from *Xylopia langsdorfian* A. St.-Hil. & Tul., the involvement of K*_ATP_* in promoting its relaxant effect on guinea pig ileum was also verified [52].

The actions of K*_ATP_* are ambiguous, and its blocking or activation can be beneficial or harmful depending on the pathology and the channel subunit expressed in the target tissue. The absence and/or dysfunction of this channel and its subunit SUR2 are implicated in increased cardiovascular risks and poor prognosis in cases of infarction and stroke, while the increase in its expression and its adequate function together bring cardio and vasoprotective effects. In neurodegenerative diseases, such as Alzheimer’s and Parkinson’s, for example, inhibition of this channel seems to be beneficial, with improvement of the underlying neuroinflammatory markers of these pathologies [53,54]. There is no consensus on the role of this channel in HUA contraction/relaxation process, and the clinical importance is still unknown relating to pregnancy-specific hypertensive diseases [51].

Many EOs are generally recognized as safe [55]. However, the use of herbs and EOs is still a highly controversial matter, and their use in clinical practice is still restricted due to their physicochemical properties (e.g., limited bioavailability) and/or their toxicity. On the other hand, they can often be excellent leads for the development of new drugs. Modifying and/or isolating the structure of these products is a strategic way to increase pharmacological action, as well as improve absorption, distribution, metabolism, and excretion properties, thus decreasing toxicity and side-effects [56]. In this context, basic research initially evaluates the pharmacological potential of such compounds, favoring decision making for clinic research aiming for their therapeutic applicability [57].

Investigating bioactive and umbilical cord artery smooth muscle opens perspectives for potential agents capable of modulating the contractile activity. The vascular smooth muscle is important for homeostasis, and several pathologies may be related to changes in vascular tone. These include hypertensive disorders, such as those afflicting pregnant women, in addition to atherosclerosis, heart failure, and ischemia [20].

We conclude that *Lippia alba* (Mill.) N.E. Brown essential oil has a vasorelaxant effect on the contractility of human umbilical artery smooth muscle. EOLa was able to inhibit contractions induced by physiological contracting agents of HUA, 5-HT and HIST, with greater potency on the pharmacomechanical coupling via the histaminergic pathway. We highlight its inhibitory action on L-type VOCCs in electromechanical coupling by K60, similar to the standard drug nifedipine. Our study also showed that K*_ATP_* channels play a discreet mediation in the relaxing effect of EOLa on HUA.

It is worth to mention that, in tissue viability investigations at the end of each experiment, full contractions were again induced by the same agonists, thus indicating that EOLa has no acute toxic effects on HUA contractility and survival. This same procedure was conducted by Carvalho et al. [17], Pereira-de-Morais et al. [19], and Silva et al. [20] using EOLa, citral, and 1-limonenedone on rodent smooth muscle, again corroborating our results of EOLa on HUA.

In conclusion, our results demonstrate the relaxing effect of EOLa on human umbilical cord arteries, encouraging further studies to be conducted on HUA using the major constituents isolated from EOLa, citral, and 1-limonene. Therefore, the hypothesis is that citral is primarily responsible for the relaxing effect of *L. alba* essential oil on HUA.

## 4. Materials and Methods

### 4.1. Solutions and Drugs

The drugs and reagents used presented analytical purity, being obtained from Sigma Chemical Corporation (St. Louis, MI, USA) or Merck (Darmstadt, Germany), and they were maintained under conditions indicated by the manufacturer. To prepare the solutions in this study, the salts used were potassium chloride (KCl), sodium chloride (NaCl), magnesium sulfate (MgSO_4_), calcium chloride (CaCl_2_), glucose (C_6_H_12_O_6_), potassium phosphate (KH_2_PO_4_), sodium carbonate (NaHCO_3_), barium chloride (BaCl_2_), ethylenediaminetetraacetic acid (EDTA), and 2-[4-(2-hydroxyethyl)piperazin-1-yl]-ethanesulfonic acid (HEPES). The concentrations were expressed in millimole/liter (mM/L).

The substances serotonin and tetraethylammonium were dissolved in distilled water, and nifedipine was diluted in ethanol; the solutions obtained were kept at 0–4 °C and only withdrawn at the moment of the experiment.

### 4.2. Lippia alba (Mill.) N.E. Brown Essential Oil

The oil, 99% pure, was purchased from Dr. Sergio Horta at the experimental farm of the Federal University of Ceará (UFC). A dried-up sample of *L. alba* species was deposited in the Prisco Bezerra Herbarium of UFC under identification code #EAC-08474.

Leaves (1 kg) were collected on the same day and hour, from flowering plants, and were steam distilled for 1 h for EO acquisition. The chemical constitution was evaluated in the Natural Products Laboratory by Dr. Afrânio Aragão Craveiro at the Technological Development Park (PADETEC) of UFC using gas chromatography coupled to mass spectrometry (GC–MS, Hewlett-Packard 6971, Harris County, TX, USA). Analysis conditions were as follows: dimethylpolysiloxane DB-1 fused silica capillary column (30 m × 0.25 mm; 0.1 μm); helium (1 mL/min) as carrier gas; injector temperature, 250 °C; detector temperature, 280 °C; column temperature, 35–180 °C at 4 °C/min and 180–250 °C at 10 °C/min; mass spectrometry electronic impact, 70 eV. The compounds were identified using mass spectral library MS searches and Kovats indices as a preselection aid [58], and visual mass spectra were compered to data from the literature for confirmation [59,60,61].

The following major constituents were encountered: citral (75.92%) (a mixture of E-geranial (41.81%) and Z-neral (34.11%)), 1-limonene (9.85%), and carvone (8.92%) (Table 2).

To analyze its activity on HUA, the EOLa was prepared as a solution, diluted directly in Krebs Henseleit with 3% Tween 80^®^ [28]. Administration was given hypertonically, analyzing the volume in each cuvette to obtain the final concentrations used inside of the organ bath chambers. A response curve was performed for EOLa concentrations of 30, 100, 200, 300, 400, 600, 800, and 1000 µg/mL.

### 4.3. Tissue Preparation and Isolation

Collection and processing of samples was approved by the Ethics Committee in Human Research of the Regional University of Cariri—URCA (nº 3.832.881), and by the Hospital and Maternity Camilo Ethics Committee. Fragments of approximately 10 cm of human umbilical cord (portions that would be destined for biological disposal), were obtained with the consent of healthy, normotensive mothers/donors and without any cord abnormalities, after childbirth (normal or cesarean). Samples were collected and stored in Krebs modified solution (composition in mM: NaCl, 125; KCl, 4.8; CaCl_2_, 1; MgSO_4_, 1.2; NaHCO_3_, 25; KH_2_PO4, 1.2; C_6_H_12_O_6_, 11; HEPES, 25; EDTA, 0.3) refrigerated and transported to the Excitable Cells Physiopharmacology Laboratory of URCA. The strand segments were stored in a refrigerator and kept at 4–8 °C, being usable for 48 h after collection [30]. The human umbilical arteries were isolated from its covering tissue, i.e., Wharton’s jelly, and cut into 3–4 mm rings.

### 4.4. Determination of Tension Exerted on the HUA Rings

After removal from Wharton’s jelly and isolation, the HUAs were sectioned into rings. In thermostated organ bath equipment, measurements were performed using a stem connected to the force transducer (MLT0420, ADInstruments Bridge Amps, ADInstruments, Sydney, Australia) which allowed capturing measurements of the isometric tension produced by the HUA rings as converted into electrical signals. The transducer was connected to an amplifier (ADInstruments Bridge Amps), and this to the input of an analog-to-digital converter board (BCN/Pod port) installed in a computer. The collected data were converted into strokes and stored in LabChart Pro software (ADInstruments) files for later analysis.

The rings were individually suspended on stainless-steel hooks inserted into their lumens and mounted using an isometric tension of 3 g. This assembly was performed in glass chambers with 10 mL of Krebs Henseleit solution, kept at 37 °C, and bubbled with a carbogenic mixture (95% O_2_; 5% CO_2_). After the artery rings were assembled, the solution was renewed every 15 min.

After a stabilization period of 90 min, all protocols began with two subsequent contractions, produced by addition of 60 mM potassium chloride (K60) in hypertonic mode to the studied HUA rings, and, after reaching stable values, the maximum response obtained was considered the maximum contraction of each ring. Then the contraction inducers KCl (60 mM) or 5-HT (10 μM) or HIST (10 µM) were added to the preparations, followed by the addition of increasing and cumulative concentrations of EOLa (30, 100, 200, 300, 400, 600, 800, and 1000 µg/mL). For each new EOLa concentration, sufficient time was allowed for the response to reach a steady state, normally 5 to 15 min. Only experiments with reproducible contractions were considered viable for the experimental series.

#### Experimental Series

*Series 1: EOLa Effect on the Basal Tone of HUA*

To evaluate the effect of EOLa on the basal HUA tone, after verification of tissue viability, a concentration–effect curve was performed by addition of increasing and cumulative concentrations of EOLa (30–1000 µg/mL) to the HUA preparations to obtain a concentration–response curve.

*Series 2: Effect of EOLa on contractions induced by KCl (60 mM), 5-HT (10 µM), and HIST (10 µM) in HUA*

To investigate the effect of EOLa on the electromechanical coupling of the HUA rings, after verifying tissue viability, contractions were induced using KCl (60 mM), and increasing and cumulative concentrations of EOLa (30–1000 µg/mL) were added to the HUA preparations to obtain a single concentration–response curve.

To evaluate the oil on pharmacomechanical coupling, we used two classical contracting agents—5-HT (10 μM) and HIST (10 μM)—one for each experiment [39]. After verification of tissue viability, contraction was induced by one of the agents, and then increasing and cumulative concentrations of EOLa (30–1000 μg/mL) were added to the HUA preparations to obtain a curve of the concentration-response.

At the end of each experiment, washings were performed for 30 consecutive minutes, and then contractions were again induced by the same agonists to demonstrate tissue viability throughout the experiment.

*Series 3: Effect of EOLa on K^+^ channels in HUA*

To evaluate the participation of K^+^ channels in this series, three blockers were used—TEA (1 mM or 10 mM), 4-AP (1 mM), or GLI (10 µM). An experiment was carried out for each blocking agent and its respective standard action concentration(s) for the subtype of K^+^ channel targeted.

TEA at a concentration of 10 mM is a nonselective potassium channel blocker, such as the high-conductance K^+^ channels activated by Ca^2+^ (BKCa). Yet, at a concentration of 1 mM, TEA selectively blocks voltage-operated K^+^ channels (K*v*). In both experiments, at their respective concentrations, TEA was added and incubated for 30 min. At the end of the incubation, the HUA rings were subjected to contractions induced by 5-HT (10 µM), and EOLa in concentrations of 30–1000 µg/mL was added to obtain a concentration-response curve.

The experiments with 4-AP and GLI, i.e., selective voltage-operated (K*v*) and ATP-sensitive K^+^ channel (K*_ATP_*) blockers, respectively, followed the protocol of the TEA series as to incubation time, contraction agent, and EOLa concentrations used.

*Series 4: Effect of EOLa on voltage-operated Ca^2+^ channels (VOCCs)*

To investigate the involvement of L-type voltage-operated calcium channels, after verifying the tissue viability, HUA preparations were kept in Krebs Henseleit zero-calcium solution incubated with EOLa (1000 µg/mL) for 30 min before receiving BaCl_2_ (0.1–30 mM). Barium ion (Ba^2+^) is a selective voltage-operated Ca^2+^ channel agonist and induces contraction depending on concentration. In this series, the control preparations, without the presence of EOLa, reached maximum contraction at 30 mM of BaCl_2_.

Nifedipine (10 µM), a dihydropyridine channel blocker, was also included as positive control and added to HUA preparations, without the presence of EOLa; after 10 min, a cumulative concentration–response curve was performed by the addition of BaCl_2_ (0.1–30 mM).

### 4.5. Statistical Analysis

Data were expressed as the mean ± SEM. Sigma Plot 11.0 software (Systat Software Inc.; San Jose, CA, USA) was used for statistical analysis and graph production. The results that were considered statistically significant presented a null hypothesis probability of less than 5% (*p* < 0.05). Student’s *t*-test and analysis of variance (one-way ANOVA) were followed (when appropriate) by Holm–Sidak and Bonferroni *t*-tests. The EC_50_ values were determined as the substance concentration able to produce 50% of the maximum effect obtained from the higher concentration used in each protocol. Normal logarithmic interpolation calculations were performed for each experiment when fitted.

The scale presented in the Y-axis graph sometimes had a broader range than the exact value used as the standard 100% contraction plateau in the stabilization period. This should be taken into account when analyzing Figure 1, where the Y-axis displays a top value of 3.0, which can be interpreted as 100% contraction made by the 3 g force upon the HUA rings. This is simply an adjustment made by the Sigma Plot 11.0 software to produce a better image for the graph, considering the value of 1 as 100% contraction obtained from the 3 g force applied to the HUA rings.

## 5. Conclusions

*Lippia alba* essential oil presents the vasorelaxant effect on human umbilical arteries, modulating both electromechanical and pharmacomechanical coupling. EOLa presents higher action via the pharmacomechanical pathway, as shown by the minor EC_50_ values recorded for both protocols using contraction agonists 5-HT and HIST, whereas its effect seems to be related mostly to the electromechanical pathway, as seen in its ability to block voltage-operated calcium channels.

## Figures and Tables

**Table 1:** Contraction agonists and blocking agents (substances); concentration values capable of triggering 50% of relaxing response (EC_50_); values of statistically significant initial concentrations (SSIC) and total relaxation (%) promoted by the *Lippia alba* essential oil in the presence of differing contractile agents and channel blockers.

Substances	EC_50_ of EOLa	SSIC, Total Relaxation (%)
Potassium chloride (60 mM)	377.0 ± 4.3 µg/mL	100 µg/mL (*p* < 0.017), 100%
Serotonin (10 µM)	339.8 ± 4.5 µg/mL	200 µg/mL (*p* < 0.001), 100%
Histamine (10 µM)	277.1 ± 8.5 µg/mL	200 µg/mL (*p* < 0.001), 100%
Tetraethylammonium (1 mM)	471.2 ± 5.8 µg/mL	200 µg/mL (*p* < 0.023), 100%
Tetraethylammonium (10 mM)	526.4 ± 4.0 µg/mL	200 µg/mL (*p* < 0.017), 100%
Glibenclamide (10 µM)	530.8 ± 6.5 µg/mL	200 µg/mL (*p* < 0.008), 90,8%
4-Aminopyridine (1 mM)	494.0 ± 8.8 µg/mL	400 µg/mL (*p* < 0.002), 100%

**Table 2:** *Lippia alba* essential oil constituents.

Constituent	Content (%)
*E*-Geranial	41.81
*Z*-Neral	34.11
Limonene	9.85
Carvone	8.92
Gamma-terpinene	2.05
Benzene-1-methyl-3-(1-methylethyl) 6-methyl-5-hepten-2-one Alpha-humulene	1.00 0.72 0.58
Linalool Beta-pinene	0.50 0.47

## Data Availability

Not applicable.
